# Mental health outcomes of ICU and non-ICU healthcare workers during the COVID-19 outbreak: a cross-sectional study

**DOI:** 10.1186/s13613-021-00900-x

**Published:** 2021-07-10

**Authors:** Hannah Wozniak, Lamyae Benzakour, Grégory Moullec, Niccolò Buetti, Alice Nguyen, Sandrine Corbaz, Pauline Roos, Laure Vieux, Jean-Claude Suard, Rafaël Weissbrodt, Jérôme Pugin, Jacques A. Pralong, Sara Cereghetti

**Affiliations:** 1 Intensive Care Unit, Geneva University Hospitals, Geneva, Switzerland; 2 Psychiatric Liaison and Crisis Intervention Service, Geneva University Hospitals, Geneva, Switzerland; 3 School of Public Health, University of Montréal, Montreal, QC Canada; 4 Infection Control Program and World Health Organization Collaborating Centre on Patient Safety, University Hospitals and Faculty of Medicine, University of Geneva, Geneva, Switzerland; 5 Personnel Health Service, Geneva University Hospitals, Geneva, Switzerland; 6 Arc School of Health, University of Applied Sciences and Arts Western Switzerland, Neuchâtel, Switzerland; 7 School of Health Sciences, University of Applied Sciences and Arts Western Switzerland, Valais-Wallis, Sion, Switzerland; 8 Faculty of Medicine, University of Geneva, Geneva, Switzerland; 9 Pulmonary Division, Geneva University Hospitals, Geneva, Switzerland

**Keywords:** Intensive care, Mental health, Caregivers, COVID-19, Healthcare worker, HCW

## Abstract

**Background:**

Intensive care workers are known for their stressful work environment and for a high prevalence of mental health outcomes. The aim of this study was to evaluate the mental health, well-being and changes in lifestyle among intensive care unit (ICU) healthcare workers (HCW) during the first wave of the COVID-19 pandemic and to compare these results with those of HCW in other hospital units. Another objective was to understand which associated factors aggravate their mental health during the COVID-19 outbreak.

**Methods:**

This cross-sectional survey collected socio-demographic data, lifestyle changes and mental health evaluations as assessed by the Generalized Anxiety Disorder 7 items (GAD-7), the Patient Health Questionnaire 9 items (PHQ-9), the Peritraumatic Distress Inventory (PDI) and the World Health Organization Well-Being Index (WHO-5) from the 28th May to 7th July 2020. The study was carried out at Geneva University Hospitals, a group of eight public hospitals in Switzerland. ICU HCW were analyzed for mental health outcomes and lifestyles changes and then compared to non-ICU HCW. A series of linear regression analyses were performed to assess factors associated with mental health scores.

**Results:**

A total of 3461 HCW were included in the study, with 352 ICU HCW. Among ICU HCW, 145 (41%) showed low well-being, 162 (46%) symptoms of anxiety, 163 (46%) symptoms of depression and 76 (22%) had peritraumatic distress**.** The mean scores of GAD-7, PHQ-9 and WHO-5 were worse in ICU HCW than in non-ICU HCW (*p* < 0.01). Working in the ICU rather than in other departments resulted in a change of eating habits, sleeping patterns and alcohol consumption (*p* < 0.01). Being a woman, the fear of catching and transmitting COVID-19, anxiety of working with COVID-19 patients, work overload, eating and sleeping disorders as well as increased alcohol consumption were associated with worse mental health outcomes.

**Conclusion:**

This study confirms the suspicion of a high prevalence of anxiety, depression, peritraumatic distress and low well-being during the first COVID-19 wave among HCW, especially among ICU HCW. This allows for the identification of associated risk factors. Long-term psychological follow-up should be considered for HCW.

**Supplementary Information:**

The online version contains supplementary material available at 10.1186/s13613-021-00900-x.

## Introduction

Over the last few years, coronaviruses have caused two major pandemics: severe acute respiratory syndrome (SARS) in 2002–2003 and Middle East respiratory syndrome MERS in 2012 [1, 2]. Since March 2020, we are facing a new global SARS-CoV-2 pandemic [2] which is having a major impact on society as well as on health care systems around the world.

This unique sanitary situation has forced many hospitals including the Geneva University Hospitals (HUG) in Switzerland to adapt to the increased flow of patients [3]. The HUG, the largest university hospital in Switzerland which comprises eight public hospitals, became a COVID-19 hospital and almost all patients with other pathologies were hospitalized in surrounding private hospitals. The capacity of certain units was increased to admit COVID-19 patients and the intensive care unit (ICU) increased its capacity from 30 to 180 beds. To strengthen the teams, healthcare workers (HCW) from other departments joined the departments most affected by the COVID-19 pandemic, such as intensive care and internal medicine departments. The HUG put in place extraordinary measures to support frontline HCW, such as a psychological support service and hotel beds close to the hospital.

During the SARS epidemic in 2003, Wu et al. showed, in the city of Beijing, China, that 10% of HCW had post-traumatic stress disorder (PTSD) and that associated risk increased threefold among staff working in close contact with SARS-positive patients [4]. More recently, studies have shown that exposure to a new infectious hazard may generate anxiety, depression or have a negative impact on quality of life [5–7]. Reorganization of work in the context of a crisis can also be a source of stress among employees [6, 8]. Moreover, psychological consequences for HCW facing a pandemic can be associated to lifestyle changes such as modified dietary and sleeping patterns as well as possible increases in alcohol or tobacco consumption [6].

ICUs are well known for their stressful work environment. Previous studies have shown that ICU nurses are at greater risk of PTSD than nurses working in other units [9] and that ICU HCW have higher levels of anxiety and depression [8, 10]. Females, nurses, direct exposure to COVID-19 patients and working in the ICU are all factors, which have been found to be associated with negative psychological impact [6, 11, 12]. Additionally, Azoulay et al*.* identified several factors associated with poorer mental health outcomes during the COVID-19 crisis, such as fear of catching the virus, inability to rest, inability to care for loved ones, emotional stress, restricted visitation for relatives of patients and having to witness hasty end-of-life decision-making [8].

In this context, we hypothesize that the COVID-19 pandemic is associated with poor mental health outcomes in HCW and especially those in the ICU. Our primary objective was to assess the mental health, well-being and changes in health behaviors of ICU HCW and to make comparisons across different professions (nurses, physicians, care assistants and others), following the first wave of the pandemic. Our secondary objectives were (1) to compare mental health outcomes between ICU and non-ICU HCW; and (2), to determine the factors associated with poor mental health and well-being. These results will help develop appropriate measures to address mental health issues of frontline HCW in case of future waves or similar outbreaks.

## Methods

### Participants and procedures

A cross-sectional survey was conducted in the HUG in Switzerland to assess levels of anxiety, depression, peritraumatic distress and well-being among HCW. A pre-tested questionnaire was sent via mailing list servers to all 13,570 HUG employees including physicians, nurses, care assistants, physiotherapists, administrative workers, maintenance workers and patient transporters. ICU HCW were defined as all the HCW who were working in the ICU during the first COVID-19 wave. This included regular ICU HCW but also HCW from other units who had to join the ICU to strengthen the teams during the pandemic. Each employee received an invitation via email to participate in the study. The email contained information on the purpose of the study and a link to the online questionnaire via the RedcapTM® platform. Participation in the study was on a voluntary basis and completely anonymous. Data were collected from May 28th, 2020, to July 7th, 2020, right at the end of the first wave of COVID-19 disease, which ended mid-May in Switzerland.

The questionnaire included questions on socio-demographic data including age, sex, nationality, country of domicile, marital status, number of children, occupation, percentage of work and type of schedule (regular/irregular). It was also assessed whether the person or one of his relatives had had the coronavirus and whether they were afraid of catching it at work or passing it on to their loved ones. The questionnaire included questions on lifestyle changes as assessed by an analog scale, including changes in alcohol consumption, smoking habits, eating patterns, levels of exercise and sleeping habits. The psychiatric scales used were the Generalized Anxiety Disorder 7 items (GAD-7), the Patient Health Questionnaire 9 items (PHQ-9) and the Peritraumatic Distress Inventory (PDI). In order to assess well-being, the World Health Organization Well-Being Index (WHO-5) was used [13–16].

The GAD-7 is a measurement tool that has been validated for screening of generalized anxiety disorder [13]. It uses 7 questions to establish the severity of anxiety and results are interpreted as follows: 0 to 4: no anxiety, 5 to 9: mild anxiety, 10 to 14: moderate anxiety, 15 to 21: severe anxiety [13]. In order to define the presence of symptoms of anxiety, we used a cut-off score ≥ 5.

The PHQ-9 is a validated score that evaluates depression through questions regarding the last 2 weeks [16]. The interpretation of the score is the following 0 to 4: no depression, 5 to 9: mild depression, 10 to 14: moderate depression, 15 to 19: moderately severe depression, 20 to 27: severe depression [16]. The presence of symptoms of depression was defined with a PHQ-9 score ≥ 5.

The PDI is a validated score that was designed to evaluate peritraumatic distress through 13 questions regarding the participant's emotional and physiological distress experienced during and immediately after a traumatic event [17, 18]. PDI ≥ 14 has been shown to predict PTSD one month after a traumatic event [14, 17], which has been defined here as the COVID-19 pandemic in Geneva.

The WHO-5 index is a screening tool based on a 5-question assessment that has been validated to assess participant well-being and has been shown to be negatively correlated with psychometric tool scores used in psychiatry to assess depression and suicidality [15, 19]. The score goes from 0 to 100 with 0 being the worst and 100 the best possible well-being score. A score below 50 speaks for low well-being [19].

This study has been approved by Geneva’s Regional Research Ethics Committee (BASEC ID 2020-00935).

### Statistical analyses

The analytical sample included ICU and non-ICU HCW who agreed to complete the questionnaire. We performed descriptive analyses of socio-demographic variables according to ICU status. For our first objective, we compared mental health (anxiety/depression symptoms and peritraumatic distress), well-being (treated as continuous scores) and changes in lifestyles (exercise, diet, sleeping habits and substance use; as categorical variables) in ICU HCW, using simple linear regression models and Chi-square tests, accordingly. Per our second objective, we compared the mental health and well-being scores and changes in lifestyle between ICU and non-ICU HCW, by performing a series of *t*-tests and Chi-square tests. Moreover, we performed a series of multivariate linear regression models with ICU status as our main independent variable. All analyses were performed using RStudio (version 1.3.1073). Two-tailed *p*-values at 0.05 were considered statistically significant. Due to the low number of missing data, a complete case analysis was applied.

## Results

### Demographic characteristics of the sample population

Of the 13,570 employees invited to take part in the study, 3461 (25%) accepted to participate. The response rate was much higher among ICU HCW with 352 answers to the survey, corresponding to a response rate of 69% (352/510), compared to 24% of the response rate among non-ICU HCW (3109/13,060) (Table 1, Additional file 1: Figure S1).

**Table 1:** Descriptive characteristics of participants (*n* = 3461)

	Total	ICU	Non-ICU
Overall	3461 (100)	352 (10)	3109 (90)
Sex			
Women, *n* (%)	2561 (74)	234 (66.5)	2327 (74.9)
Men, *n* (%)	897 (26)	118 (33.5)	779 (25.1)
Age			
18–29 years, *n* (%)	402 (11.6)	39 (11)	363 (11.7)
30–39 years, *n* (%)	815 (23.6)	130 (36.9)	685 (22)
40–49 years, *n* (%)	1032 (29.8)	97 (27.6)	935 (30.1)
50–59 years, *n* (%)	1049 (30.3)	75 (21.3)	974 (31.3)
≥ 60 years, *n* (%)	163 (4.7)	11 (3.1)	152 (4.9)
Marital status			
Single, *n* (%)	767 (22.2)	85 (24.2)	682 (21.9)
Married, *n* (%)	2215 (64)	242 (68.7)	1973 (63.5)
Divorced, *n* (%)	451 (13)	25 (7.1)	426 (13.7)
Widow(-er), *n* (%)	27 (0.8)	0 (0)	27 (0.9)
Minor dependent children			
Yes, *n* (%)	1635 (47.2)	185 (52.6)	1450 (46.6)
No, *n* (%)	1826 (52.8)	167 (47.4)	1659 (53.4)
Profession			
Physician, *n* (%)	438 (12.7)	68 (19.3)	370 (11.9)
Nurse, *n* (%)	1341 (38.8)	198 (56.3)	1143 (36.8)
Care assistant, *n* (%)	261 (7.5)	32 (9.1)	229 (7.4)
Others, *n* (%)	1420 (41)	54 (15.3)	1366 (43.9)
Employment rate			
Before the pandemic, %(SD)	86 (17)	87.5 (16)	85.8 (17.1)
During the pandemic, %(SD)	87 (16.2)	90.1 (15)	86.6 (16.3)
Schedule change during the pandemic			
Yes, *n* (%)	1949 (56.4)	297 (84.4)	1652 (53.2)
No, *n* (%)	1507 (43.6)	55 (15.6)	1452 (46.7)
Change in workload during the pandemic			
Less workload than usual, *n* (%)	512 (14.8)	60 (17.1)	452 (14.6)
Overload, *n* (%)	875 (25.3)	104 (29.6)	771 (24.8)
Same workload as usual, *n* (%)	2067 (59.8)	187 (53.3)	1880 (60.6)
Country of residence			
Switzerland, *n* (%)	1927 (56)	167 (47.6)	1760 (56.9)
France, *n* (%)	1516 (44)	184 (52.4)	1332 (43.1)
Relatives who have had COVID-19 disease			
Yes, *n* (%)	833 (24.1)	68 (19.3)	765 (24.6)
No, *n* (%)	2628 (75.9)	284 (80.7)	2344 (75.4)
Fear of catching COVID-19 disease			
Yes, *n* (%)	779 (22.5)	75 (21.3)	704 (22.6)
Rather yes, *n* (%)	892 (25.8)	90 (25.6)	802 (25.8)
Rather no, *n* (%)	1164 (33.6)	122 (34.7)	1042 (34.5)
No, *n* (%)	626 (18.1)	65 (18.4)	561 (18.1)
Fear of transmitting COVID-19 disease			
Yes, *n* (%)	1592 (46.1)	195 (55.4)	1397 (45)
Rather yes, *n* (%)	1017 (29.4)	94 (26.7)	923 (29.7)
Rather no, *n* (%)	553 (16)	42 (11.9)	511 (16.5)
No, *n* (%)	293 (8.5)	21 (6)	272 (8.8)
Fear of working with COVID-19 patients			
Yes, *n* (%)	682 (19.7)	112 (31.8)	570 (18.3)
Rather yes, *n* (%)	1097 (31.7)	120 (34.1)	977 (31.4)
Rather no, *n* (%)	846 (24.4)	99 (28.1)	747 (24.1)
No, *n* (%)	836 (24.2)	21 (6)	815 (26.2)
Use of any psychological support			
Yes, *n* (%)	420 (12.1)	98 (27.8)	322 (10.4)
No, *n* (%)	3041 (77.9)	254 (72.2)	2787 (89.6)
Hotel accommodation during the pandemic			
Yes, *n* (%)	231 (6.7)	84 (24.9)	147 (4.7)
No, *n* (%)	3230 (93.3)	268 (76.1)	2962 (95.3)

Values were expressed in numbers and percentages

Seventy-four percent of participants were women (2561/3461) and age ranged from 18 to 65 years. Seven-hundred and sixty-seven (22.2%) participants were single and 2215 (64%) were married. Regarding occupational categories 1341 (38.8%) participants were nurses, 438 (12.7%) were physicians, 261 (7.5%) were care assistants and 1420 (41%) had other functions such as administrative workers. Among the study population, 1949 (56.4%) reported having had changes in their schedule during the pandemic and 875 (25.3%) reported being overworked. Regarding coronavirus issues, 833 (24.1%) participants reported having a relative who had been infected with SARS-CoV-2, 779 (22.5%) feared getting infected with SARS-CoV-2 and 1592 (46.1%) were afraid of transmitting it. Further details are illustrated in Table 1.

### Prevalence of anxiety, depression, peritraumatic distress and low well-being for ICU HCW

352 ICU HCW including 198 (56%) nurses, 68 (19%) physicians, 54 (15%) other workers and 32 (9%) care assistants responded to the questionnaire on psychiatric assessment (Table 2). The average GAD-7 score was 6.1 (SD 4.8), indicating mild anxiety, with 25 (7.1%) of the GAD-7 score reflecting severe anxiety and 49 (13.9%) moderate anxiety. The average PHQ-9 score was 6.4 (SD 5) reflecting mild depression, with 6 (1.7%) of the population having a score predicting severe depression, 24 (6.8%) moderate-to-severe depression and 54 (15.3%) moderate depression. No statistical differences were found in the symptoms of anxiety and depression by occupational category. Regarding PDI, the mean score was 8.8 (SD 7.4) and 76 (21.6%) of the ICU HCW had a score ≥ 14 that put them at risk of developing PTSD at one month. The PDI was statistically different by occupational category with a mean score of 8.4 (SD 7.7) for physicians, 9 (SD 7.3) for nurses, 11.7 (SD 8.9) for care assistants, 6.7 (SD 5.6) for other HCW. The average WHO-5 score was 53.3 (SD 23.8) with 145 (41.2%) of the population having a WHO-5 below 50 reflecting low well-being. The WHO-5 score was statistically different by occupational category with a mean score of 56.8 (SD 21.8) for physicians, 50.2 (SD 25.2) for nurses, 55 (SD 23) for care assistants, 59.3 (SD 19.6) for other HCW.

**Table 2:** Score descriptions for ICU HCW (*n* = *352*)

	ICU (*n* = 352)					*p* value
	Total	Physicians	Nurses	Care assistants	Others	
Overall, *n* (%)	352 (100)	68 (19)	198 (56)	32 (9)	54 (15)	
GAD-7						
Mean score (SD)	6.1 (4.8)	6.0 (5.0)	6.3 (5.0)	6.5 (4.7)	5.0 (4.1)	0.33
Minimal anxiety, *n* (%)	190 (54)	41 (60.3)	98 (49.5)	14 (43.7)	37 (68.5)	0.32
Mild anxiety, *n* (%)	88 (25)	12 (17.7)	55 (27.8)	11 (34.4)	10 (18.5)	
Moderate anxiety, *n* (%)	49 (13.9)	10 (14.7)	30 (15.1)	4 (12.5)	5 (9.3)	
Severe anxiety, *n* (%)	25 (7.1)	5 (7.3)	15 (7.6)	3 (9.4)	2 (3.7)	
PHQ-9						
Mean score (SD)	6.4 (5.0)	6.4 (5.5)	6.7 (5.2)	6.8 (4.9)	5.1 (3.8)	0.20
Minimal depression, *n* (%)	189 (53.7)	40 (58.8)	97 (49)	16 (50)	36 (66.7)	0.34
Mild depression, *n* (%)	79 (22.4)	12 (17.7)	47 (23.7)	8 (25)	12 (22.2)	
Moderate depression, *n* (%)	54 (15.3)	10 (14.7)	35 (17.7)	5 (15.6)	4 (7.4)	
Moderately severe depression, *n* (%)	24 (6.8)	3 (4.4)	16 (8.1)	3 (9.4)	2 (3.7)	
Severe depression, *n* (%)	6 (1.7)	3 (4.4)	3 (1.5)	0 (0)	0 (0)	
PDI						
Mean score (SD)	8.8 (7.4)	8.4 (7.7)	9.0 (7.3)	11.7 (8.9)	6.7 (5.6)	0.02
Not a risk for PTSD, *n* (%)	276 (78.4)	56 (82.4)	151 (76.3)	21 (66)	48 (88.9)	0.05
At risk for PTSD, *n* (%)	76 (21.6)	12 (17.6)	47 (23.7)	11 (34)	6 (11.1)	
WHO-5						
Mean score (SD)	53.3 (23.8)	56.8 (21.8)	50.2 (25.2)	55.0 (23.0)	59.3 (19.6)	0.03
< 50, *n* (%)	145 (41.2)	22 (32)	95 (48)	14 (44)	14 (26)	0.01
≥ 50 *n* (%)	207 (58.8)	46 (68)	103 (52)	18 (56)	40 (74)	

Values were expressed in numbers and percentages; mean values and standard deviation

*WHO-5* World Health Organization Well-Being Index, *GAD-7* 7-item Generalized Anxiety Disorders, *PHQ-9* 9 items Patient Health Questionnaire, *PDI* Peritraumatic Distress Inventory, *NA* not available

### Lifestyle changes descriptions for ICU HCW

Regarding lifestyle factors among ICU HCW (Table 3), 159 respondents (45.2%) reported sleeping less than usual, 114 (32.4%) eating more. Concerning physical exercise, 164 (46.6%) reported doing less sport than usual. Considering alcohol and tobacco consumption, 78 (22.2%) respondents reported an increase in alcohol consumption and 53 (15%) reported an increase in tobacco consumption. No statistically significant differences were shown in the different occupational categories for lifestyle changes.

**Table 3:** Lifestyle changes descriptions for ICU HCW (*n* = 352)

	ICU (*n* = 352)					*p* value
	Total	Physicians	Nurses	Care assistants	Others	
Overall, *n* (%)	352 (100)	68 (19)	198 (56)	32 (9)	54 (15)	
Sleeping habits						0.26
Less than usual, *n* (%)	159 (45.2)	24 (35.3)	95 (48)	13 (40.6)	27 (50)	
Same as usual, *n* (%)	95 (27)	26 (38.2)	48 (24.2)	7 (21.9)	14 (25.9)	
More than usual, *n* (%)	98 (27.8)	18 (26.5)	55 (27.8)	12 (37.5)	13 (24.1)	
Eating habits						0.21
Less than usual, *n* (%)	47 (13.4)	5 (7.4)	28 (14.1)	7 (21.9)	7 (12.9)	
Same as usual, *n* (%)	191 (54.2)	41 (60.3)	100 (50.6)	20 (62.5)	30 (55.6)	
More than usual, *n* (%)	114 (32.4)	22 (32.3)	70 (35.3)	5 (15.6)	17 (31.5)	
Exercise						0.07
Less than usual, *n* (%)	164 (46.6)	30 (44.1)	103 (52)	12 (37.5)	19 (35.2)	
Same as usual, *n* (%)	131 (37.2)	22 (32.4)	71 (35.9)	12 (37.5)	26 (48.1)	
More than usual, *n* (%)	57 (16.2)	16 (23.5)	24 (12.1)	8 (25)	9 (16.7)	
Alcohol						0.06
Less than usual, *n* (%)	29 (8.2)	3 (4.4)	16 (8.1)	5 (15.6)	5 (9.3)	
Same as usual, *n* (%)	245 (69.6)	44 (64.7)	135 (68.2)	25 (78.1)	41 (75.9)	
More than usual, *n* (%)	78 (22.2)	21 (30.9)	47 (23.7)	2 (6.3)	8 (14.8)	
Tobacco						0.16
Less than usual, *n* (%)	14 (4)	3 (4.4)	7 (3.5)	3 (9.4)	1 (1.8)	
Same as usual, *n* (%)	285 (81)	55 (80.9)	158 (79.8)	22 (68.7)	50 (92.6)	
More than usual, *n* (%)	53 (15)	10 (14.7)	33 (16.7)	7 (21.9)	3 (5.6)	

Values were expressed in numbers and percentages

### Comparison of mental health outcomes, well-being and lifestyle changes between ICU HCW and non-ICU HCW

**Fig. 1:**
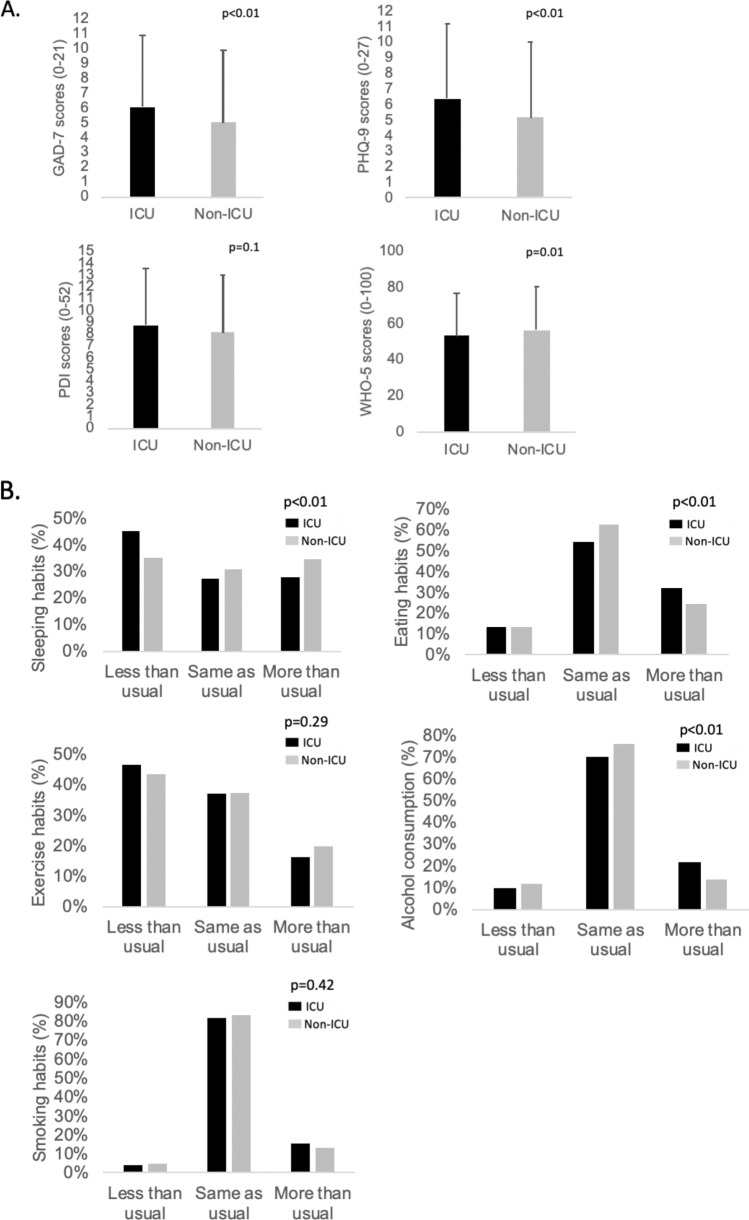
Comparison of psychiatric scores, well-being and lifestyle changes between ICU and non-ICU HCW. **A** Comparison of psychiatric scores and well-being between ICU and non-ICU HCW. **B** Comparison of lifestyle changes between ICU and non-ICU HCW

Figure 1 compares ICU and non-ICU HCW regarding mental health, well-being and lifestyle changes. The mean GAD-7 and PHQ-9 scores were higher in ICU compared to non-ICU HCW, while ICU HCW reported lower well-being scores (WHO-5). No significant difference in PDI score was observed. In terms of lifestyle habits, working in the ICU rather than in another hospital unit was associated with having a change in diet (*p* < 0.01), in sleeping patterns (< 0.01) and in alcohol consumption (*p* < 0.01). Details can be found in Additional file 1: Table S1.

### Identification of risk factors of poor mental health outcome and low well-being among ICU and non-ICU HCW

Table 4 presents the factors that have been found to be associated with an increase in GAD-7, PHQ-9 and PDI score and a decrease in the WHO-5 scale among all the employees (ICU HCW and non-ICU HCW). We were able to identify common factors associated with the 4 scores that we can classify into several categories: (1) socio-demographic factors: being female; (2) working environmental factors: being overloaded with work; (3) anxiety towards COVID-19: fear of catching and transmitting it, anxiety of working in contact with COVID-19 patients; (4) somatic symptoms: eating less, sleeping disorders; (5) impact on consumptions: drinking more alcohol. Details of multivariable models are available in the Additional file 1: Table S2.

**Table 4:** Identification of risk factors of high psychiatric scores and low well-being scale among ICU and non-ICU HCW

GAD-7	PHQ-9	PDI	WHO-5
Female No minor children Work overload Fear of catching COVID-19 Fear of transmitting COVID-19 Stress of working in Contact with COVID-19 Trouble sleeping Eating less More alcohol	Older Female Single Work overload Fear of catching COVID-19 Fear of transmitting COVID-19 Stress of working in contact with COVID-19 Trouble sleeping Eating less More alcohol Less physical exercise	Older Female Single Work overload Care assistant Relatives affected by COVID-19 Fear of catching COVID-19 Fear of transmitting COVID-19 Stress of working in contact with COVID-19 Trouble sleeping Eating less More alcohol	Female Single Work overload Occupation Relatives affected by COVID-19 Fear of catching COVID-19 Fear of transmitting COVID-19 Stress of working in contact with COVID -19 Trouble sleeping Eating less More alcohol Less physical exercise

*WHO-5* World Health Organization Well-Being Index, *GAD-7* 7-item Generalized Anxiety Disorders, *PHQ-9* 9 items Patient Health Questionnaire, *PDI* Peritraumatic Distress Inventory

## Discussion

This cross-sectional study included a total of 3,461 HCW of which 352 were ICU HCW during the first wave of the COVID-19 crisis. Among ICU HCW, 41% had low well-being, 46% had anxiety symptoms, 46% had symptoms of depression and 22% had peritraumatic distress. Scores for risk of depression, anxiety and low well-being were statistically more pathological in the ICU than in other hospital units. A change in lifestyle factors was also highlighted with an increase in alcohol consumption and a modification of eating and sleeping habits among ICU HCW. In the entire studied population (ICU and non-ICU HCW), several factors were found to be associated with symptoms of anxiety, depression, peritraumatic distress and low well-being: being female, the fear of catching and transmitting COVID-19, anxiety of working in contact with COVID-19 patients, being overloaded with work, eating less, increased alcohol consumption and sleeping disorders.

With regard to anxiety and depression among ICU HCW, the present study confirms the findings of several studies conducted during the pandemic reporting a prevalence of anxiety ranging from 48 to 50.4% and depression ranging from 16 to 30.4% among ICU HCW [6, 8]. Comparison with these studies’ results should be made with caution as different scores were used. Many factors may explain the high prevalence of depression and anxiety symptoms described in ICU HCW during this crisis. Firstly, the high rate of anxiety could be explained by the fact that COVID-19 appeared unpredictable and potentially lethal. HCW were exposed to this uncertainty which not only concerned their patients, but themselves as well, making them feel powerless. Secondly, the media coverage of the events with the announcement in February 2020 of more than 3000 caregivers infected with COVID-19 in China could have been a source of stress for HCW [20]. Thirdly, during this first wave of COVID-19, the HUG mainly treated COVID-19 patients while patients with other pathologies were hospitalized in the surrounding hospitals. This distribution could have been a source of anxiety and depression for HCW who were continuously exposed to this extreme situation. No differences in the symptoms of anxiety and depression by occupational category were highlighted in our study. This might be explained by the fact that in our hospital, the staff that had been widely recruited has made it possible to maintain good working conditions with an average of one nurse for every two patients in the ICU. Studies [6, 11] describing a more important role of anxiety and depression in nurses did not mention this type of information thus making this interpretation harder to ascertain.

We assessed peritraumatic distress in ICU HCW because we suspected that the COVID-19 pandemic would potentially expose them to traumatic events [21], such as numerous and unpredictable deaths [22]. Our study showed that 22% of ICU HCW displayed peritraumatic distress. Peritraumatic distress, which is defined as the emotional and physiological distress experienced during and immediately after a traumatic event, is a known risk factor of developing PTSD one month after a traumatic event [14]. Indeed, a French study observed that 27% of ICU HCW experienced PTSD in the context of the pandemic [6]. These results highlight the important risk of PTSD for ICU HCW and the need to implement preventative measures to support them. Protective factors for PTSD include good coping strategies in stressful situations, primary prevention, training before a traumatic event and positive social support following a traumatic event [23, 24].

Another objective of our study was to assess whether ICU HCW had suffered more from the pandemic compared to non-ICU HCW. Indeed, we showed that ICU HCW had more symptoms of anxiety, depression and lower well-being. The ICU is known to be a difficult work environment due to heavy workloads, exposure to critically ill patients, daily confrontation with death and irregular working hours [10]. Even outside major crises, ICU HCW have been shown to be more prone to anxiety and depression compared to staff from other units [10, 25]. During catastrophic situations, ICU HCW tend to leave their needs aside to meet the needs of patients [6]. In an already stressful work environment, poorer mental health outcomes can be expected to be exacerbated by the stress caused by the pandemic with many unknowns, the fear of catching and transmitting the virus, the high influx of patients, the fear of not having enough resources and changes in work habits. However, unlike what might have been expected, no significant difference was found in peritraumatic distress between ICU HCW and non-ICU HCW. As the exposition to traumatic events of unexpected and numerous deaths occurred in all hospital units and not only in the ICU, this could explain the lack of difference in peritraumatic distress between the two groups. A feature of our study was to include a significant rate of “other workers” (41% of our total sample), such as administrative workers, while other studies disregarded this population. Interestingly, a sensitivity analysis showed that this group of workers [over-represented in non-ICU (44%) vs. ICU (15%)] did not influence the differences observed in mental health and well-being outcomes between ICU and non-ICU HCW.

The present study showed important changes in lifestyle behavior in HCW and ICU HCW appeared to have increased their alcohol consumption more than non-ICU HCW. Studies have shown that exposure to traumatic events, such as terrorist attacks, natural events or in this case the COVID-19 outbreak, is associated with increased alcohol consumption [26]. This has also been shown in the general population where an increase in stress-related alcohol consumption during the COVID-19 pandemic has been highlighted [27]. To the best of our knowledge, we are the first to raise this point in HCW in the context of the pandemic and to show its association with more anxiety, depression, peritraumatic distress and low well-being in this setting. The potential for alcohol abuse by exposed HCW must be recognize as it may have implications for their physical health and should be followed up.

Another objective was to identify risk factors for worse mental health outcomes in all the HCW (ICU and non-ICU). Our study significantly showed that ICU HCW suffered more psychologically than non-ICU HCW. Interestingly, after adjustment for socio-demographic variables and lifestyle behaviors, working in the ICU was no longer an independent predictor of poor mental health outcomes. However, we were able to identify several independent risk factors for poor mental health outcomes in all the HCW (ICU and non-ICU). These factors were classified into 5 categories. (1) socio-demographic factors: female have been found to have more anxiety, depression and peritraumatic distress in our study, which is an already known risk factor even outside the coronavirus outbreak [5, 7, 11]. (2) Working environmental factors: as previously described in a French study, being overworked was associated with poorer mental health outcome [28]. A strategy that allows for break times and balanced work schedule could help control this factor. (3) Somatic symptoms: sleep disorders and changes in eating habits among HCW were associated with poor mental health outcomes in the context of the COVID-19 crisis. Indeed, several studies have identified sleeping disorders in frontline HCW during the pandemic [11, 29]. (4) Consumptions: our results show that increased alcohol consumption has been found to be independently associated with poor mental health outcome. (5) Fear towards COVID-19: we were able to identify specific risk factors in the context of this pandemic and found that being afraid of catching, transmitting or working with COVID-19 patients was associated with higher anxiety, depression and peritraumatic distress. As raised in the Azoulay et al. study, fear leads to general discomfort, fatigue and difficulty in decision-making [8]. Since the feeling of fear among HCW was frequently reported in several studies, providing regular, accurate and detailed information on the virus, its mode of transmission and associated protective measures, seems crucial [6, 8, 20]. This study reinforces the knowledge on factors associated with poor mental health outcomes during the COVID-19 crisis. In light of these considerations, hospital managers should be able to pay particular attention to HCW at risk [8, 11, 12, 29].

Some limitations in our study need to be acknowledged. The cross-sectional design of our study does not allow us to infer causality between the factors studied and the psychic symptoms, but only to find an association. Only 25% of the total HCW responded to the study questionnaire and a selection bias is therefore possible; however, the response rate was high among ICU HCW (69%). Another limitation was our inability to assess psychiatric history or previous trauma. The study was monocentric; however, the HUG is a large hospital consortium comprising eight different sites. Finally, we did not further assess ICU HCW to differentiate between back-up ICU HCW and usual ICU HCW.

## Conclusion

Our results strengthen the findings of previous studies conducted on the mental health of HCW during the COVID-19 outbreak and highlight the high prevalence of anxiety, depression, peritraumatic distress and low well-being, especially among ICU HCW. Lifestyle changes in areas such as amount of physical activity, sleeping and eating patterns as well as alcohol and tobacco consumption were also highlighted during the pandemic. Long-term psychological follow-up should be considered for HCW.

## Supplementary Information

**Additional file 1: Table S1.** Comparison of psychiatric scores, well-being and lifestyle changes between ICU and non-ICU HCW. **Table S2.** Factors associated with mental health and well-being outcomes. **Figure S1.** Flowchart of survey population sampling.

## Data Availability

The datasets used and analyzed during the current study are available from the corresponding author on reasonable request.
