# Supplementary material for: Mental health outcomes of ICU and non-ICU healthcare workers during the COVID-19 outbreak: a cross-sectional study
Source: Ann Intensive Care. 2021 Jul 10;11:106. doi: 10.1186/s13613-021-00900-x (PMC8271328; doi:10.1186/s13613-021-00900-x)
Supplement: Supplementary file 1 — Additional file 1: Table S1. Comparison of psychiatric scores, well-being and lifestyle changes between ICU and non-ICU HCW. Table S2. Factors associated with mental health and well-being outcomes. Figure S1. Flowchart of survey population sampling. [file 13613_2021_900_MOESM1_ESM.docx]

**Table S1.** Comparison of psychiatric scores, well-being and lifestyle changes between ICU and non-ICU HCW

|  | ICU | | Non-ICU | | p value |
| --- | --- | --- | --- | --- | --- |
| Overall | 352 |  | 3109 |  |  |
| GAD-7 |  |  |  |  |  |
| mean (SD) | 6,1 | (4,8) | 5,0 | (4,5) | *<0.01* |
| PHQ-9 |  |  |  |  |  |
| mean (SD) | 6,4 | (5,0) | 5,2 | (4,8) | *<0.01* |
| PDI |  |  |  |  |  |
| mean (SD) | 8,8 | (7,4) | 8,1 | (6,9) | *0.1* |
| WHO-5 |  |  |  |  |  |
| mean (SD) | 53,3 | (23,8) | 56,6 | (23,0) | *0.01* |
| Sleeping habits |  |  |  |  | *<0.01* |
| Less than usual, n(%) | 159 | (45.2) | 1091 | (35.1) |  |
| Same as usual, n(%) | 95 | (27) | 944 | (30.4) |  |
| More than usual, n(%) | 98 | (27.8) | 1074 | (34.5) |  |
| Eating habits |  |  |  |  | *<0.01* |
| Less than usual, n(%) | 47 | (13.4) | 408 | (13.1) |  |
| Same as usual, n(%) | 191 | (54.2) | 1943 | (62.5) |  |
| More than usual, n(%) | 114 | (32.4) | 758 | (24.4) |  |
| Exercise |  |  |  |  | *0.29* |
| Less than usual, n(%) | 164 | (46.6) | 1349 | (43.4) |  |
| Same as usual, n(%) | 131 | (37.2) | 1158 | (37.2) |  |
| More than usual, n(%) | 57 | (16.2) | 602 | (19.4) |  |
| Alcohol |  |  |  |  | *<0.01* |
| Less than usual, n(%) | 29 | (8.2) | 346 | (11.1) |  |
| Same as usual, n(%) | 245 | (69.6) | 2350 | (75.6) |  |
| More than usual, n(%) | 78 | (22.2) | 413 | (13.3) |  |
| Tobacco |  |  |  |  | *0.42* |
| Less than usual, n(%) | 14 | (4) | 131 | (4.2) |  |
| Same as usual, n(%) | 285 | (81) | 2579 | (83) |  |
| More than usual, n(%) | 53 | (15) | 399 | (12.8) |  |

Abbreviations: WHO-5, World Health Organization Well-Being Index. GAD-7, 7-item Generalized Anxiety Disorders. PHQ-9, 9 items Patient Health Questionnaire. PDI, Peritraumatic Distress Inventory.

Legend: Values were expressed in numbers and percentages; mean values and standard deviation

**Table S2.** Factors associated with mental health and well-being outcomes

|  | PDI | | | | GAD-7 | | | | PHQ-9 | | | | WHO-5 | | | |
| --- | --- | --- | --- | --- | --- | --- | --- | --- | --- | --- | --- | --- | --- | --- | --- | --- |
|  | Beta coef. | (95%CI) | | *p value* | Beta coef. | (95%CI) | | *p value* | Beta coef. | (95%CI) | | *p value* | Beta coef. | (95%CI) | | *p value* |
| Age |  |  |  |  |  |  |  |  |  |  |  |  |  |  |  |  |
| 18-29 years old | -1.41 | (-2.15 to -0.68) | | **<0.01** | -0.17 | (-0.64 to 0.31) | | 0.48 | -0.56 | (-1.07 to -0.04) | | **0.03** | 1.79 | (-0.61 to 4.19) | | 0.91 |
| 30-39 years old | -1.19 | (-1.76 to -0.62) | | **<0.01** | -0.41 | (-0.78 to -0.04) | | **0.03** | -0.47 | (-0.87 to -0.08) | | **0.02** | 0.32 | (-1.55 to 2.18) | | 0.14 |
| 40-49 years old | -0.83 | (-1.36 to -0.3) | | **<0.01** | -0.14 | (0.48 to 0.20) | | 0.42 | -0.03 | (-0.40 to 0.34) | | 0.89 | -1.20 | (-2.93 to 0.53) | | 0.74 |
| 50-59 years old | Ref. | | | | Ref. | | | | Ref. | | | | Ref. | | | |
| ≥ 60 years old | 0.65 | (-0.29 to 1.58) | | 0.18 | 0.26 | (-0.35 to 0.87) | | 0.84 | 0.42 | (-0.23 to 1.07) | | 0.21 | 0.28 | (-2.79 to 3.34) | | 0.86 |
| Sex |  |  |  |  |  |  |  |  |  |  |  |  |  |  |  |  |
| Women | Ref. | | | | Ref. | | | | Ref. | | | | Ref. | | | |
| Men | -1.07 | (-1.51 to -0.62) | | **<0.01** | -0.85 | (-1.13 to -0.56) | | **0.00** | -0.83 | (-1.14 to -0.52) | | **<0.01** | 4.53 | (3.08 to 5.98) | | **<0.01** |
| Country of residence |  |  |  |  |  |  |  |  |  |  |  |  |  |  |  |  |
| Switzerland |  |  |  |  |  |  |  |  |  |  |  |  |  |  |  |  |
| France | -0.25 | (-0.68 to 0.18) | | 0.25 | -0.02 | (-0.30 to 0,26) | | 0.88 | 0.07 | (-0.23 to 0.37) | | 0.64 | -0.61 | (-2.01 to 0.80) | | 0.40 |
| Other | 1.32 | (-1.76 to 4.41) | | 0.40 | 1.28 | (-0.71 to 3.27) | | 0.21 | 0.07 | (-20.7 to 2.22) | | 0.95 | -2.41 | (-12.47 to 7.65) | | 0.64 |
| Marital status |  |  |  |  |  |  |  |  |  |  |  |  |  |  |  |  |
| Single | 0.93 | (0.37 to 1.48) | | **<0.01** | 0.09 | (-0.27 to 0.45) | | 0.63 | 0.72 | (0.33 to 1.10) | | **0.00** | -2.97 | (-4.78 to -1.16) | | **<0.01** |
| Married | Ref. | | | | Ref. | | | | Ref. | | | | Ref. | | | |
| Divorced | -0.45 | (-1.04 to 0.14) | | 0.13 | -0.52 | (-0.90 to -0.14) | | **0.01** | -0.29 | (-0.70 to 0.12) | | 0.17 | 0.32 | (-1.61 to 2.24) | | 0.75 |
| Widow(-er) | 0.68 | (-1.47 to 2.83) | | 0.53 | 0.00 | (-1.39 to 1.39) | | 1.00 | 0.88 | (-0.61 to 2.37) | | 0.25 | -2.26 | (-9.27 to 4.75) | | 0.53 |
| Minor dependent children |  |  |  |  |  |  |  |  |  |  |  |  |  |  |  |  |
| Yes | Ref. | | | | Ref. | | | | Ref. | | | | Ref. | | | |
| No | 0.03 | (-0.44 to 0.5) | | 0.89 | 0.34 | (0.03 to 0.64) | | **0.03** | -0.13 | (-0.46 to 0.19) | | 0.42 | 1.49 | (-0.03 to 3.02) | | 0.06 |
| Hotel Accommodation during the Pandemic |  |  |  |  |  |  |  |  |  |  |  |  |  |  |  |  |
| Yes | Ref. | | | | Ref. | | | | Ref. | | | |  |  |  |  |
| No | 0.65 | (-0.15 to 1.44) | | 0.11 | 0.05 | (-0.46 to 0.56) | | 0.85 | 0.07 | (-0.48 to 0.62) | | 0.80 | -3.03 | (-5.62 to -0.44) | | **0.02** |
| Change in workload during the pandemic |  |  |  |  |  |  |  |  |  |  |  |  |  |  |  |  |
| Less workload than usual | -0.08 | (-0.63 to 0.46) | | 0.76 | -0.22 | (-0.58 to 0.13) | | 0.21 | 0.14 | (-0.24 to 0.52) | | 0.46 | 0.92 | (-0.87 to 2.71) | | 0.31 |
| Overload | 0.52 | (0.07 to 0.98) | | **0.02** | 0.62 | (0.33 to 0.92) | | **<0.01** | 0.63 | (0.31 to 0.94) | | **<0.01** | -3.00 | (-4.49 to -1.51) | | **<0.01** |
| Same workload as usual | Ref. | | | | Ref. | | | | Ref. | | | | Ref. | | | |
| Work department |  |  |  |  |  |  |  |  |  |  |  |  |  |  |  |  |
| Other | Ref. | | | | Ref. | | | | Ref. | | | | Ref. | | | |
| ICU | -0.16 | (-0.82 to 0.50) | | 0.64 | 0.30 | (-0.13 to 0.72) | | 0.17 | 0.39 | (-0.07 to 0.85) | | 0.09 | 0.13 | (-2.03 to 2.29) | | 0.91 |
| Profession |  |  |  |  |  |  |  |  |  |  |  |  |  |  |  |  |
| Physician | Ref. | | | | Ref. | | | | Ref. | | | | Ref. | | | |
| Nurse | 0.06 | (-0.71 to 0.73) | | 0.86 | -0.41 | (-0.85 to 0.03) | | 0.07 | -0.23 | (-0.70 to 0.24) | | 0.34 | 2.15 | (-0.06 to 4.35) | | 0.06 |
| Care assistant | 1.06 | (0.15 to 1.98) | | **0.02** | -0.16 | (-0.75 to 0.43) | | 0.60 | 0.05 | (-0.58 to 0.69) | | 0.87 | 4.06 | (1.08 to 7.05) | | **0.01** |
| Others | 0.04 | (-0.71 to 0.61) | | 0.84 | -0.29 | (-0.71 to 0.14) | | 0.18 | -0.18 | (-0.64 to 0.28) | | 0.44 | 2.62 | (0.47 to 4.78) | | **0.02** |
| Relatives who have had COVID-19 disease |  |  |  |  |  |  |  |  |  |  |  |  |  |  |  |  |
| Yes | 0.59 | (0.15 to 1.04) | | **<0.01** | 0.26 | (-0.02 to 0.55) | | 0.07 | 0.29 | (-0.02 to 0.60) | | 0.07 | -1.69 | (-3.14 to -0.23) | | **0.02** |
| No | Ref. | | | | Ref. | | | | Ref. | | | | Ref. | | | |
| Fear of catching COVID-19 disease |  |  |  |  |  |  |  |  |  |  |  |  |  |  |  |  |
| Yes | Ref. | | | | Ref. | | | | Ref. | | | | Ref. | | | |
| Rather yes | -2.42 | (-3 to -1.83) | | **<0.01** | -1.45 | (-1.83 to -1.07) | | **<0.01** | -0.99 | (-1.40 to -0.59) | | **<0.01** | 3.85 | (1.93 to 5.76) | | **<0.01** |
| Rather no | -3.41 | (-4.03 to -2.79) | | **<0.01** | -2.22 | (-2.62 to -1.82) | | **<0.01** | -1.18 | (-1.61 to -0.75) | | **<0.01** | 6.22 | (4.20 to 8.24) | | **<0.01** |
| No | -4.29 | (5.05 to -3.54) | | **<0.01** | -2.45 | (-2.94 to -1.96) | | **<0.01** | -1.42 | (-1.95 to-0.89) | | **<0.01** | 10.14 | (7.66 to 12.63) | | **<0.01** |
| Fear of transmitting COVID-19 disease |  |  |  |  |  |  |  |  |  |  |  |  |  |  |  |  |
| Yes | Ref. | | | | Ref. | | | | Ref. | | | | Ref. | | | |
| Rather yes | -1.8 | (-2.3 to -1.3) | | **<0.01** | -0.59 | (-0.91 to -0.27) | | **<0.01** | -0.61 | (-0.96 to -0.27) | | **<0.01** | 3.79 | (2.16 to 5.41) | | **<0.01** |
| Rather no | -2.64 | (-3.28 to -2) | | **<0.01** | -0.94 | (-1.35 to -0.53) | | **<0.01** | -0.91 | (-1.36 to -0.47) | | **<0.01** | 5.70 | (3.61 to 7.80) | | **<0.01** |
| No | -2.76 | (-3.66 to -3.53) | | **<0.01** | -0.88 | (-1.42 to -0.34) | | **<0.01** | -1.02 | (-1.60 to -0.44) | | **<0.01** | 7.21 | (4.48 to 9.94) | | **<0.01** |
| Fear of working with COVID-19 patients |  |  |  |  |  |  |  |  |  |  |  |  |  |  |  |  |
| Yes | Ref. | | | | Ref. | | | | Ref. | | | |  |  |  |  |
| Rather yes | -3.07 | (-3.66 to -2.47) | | **<0.01** | -1.71 | (-2.09 to -1.33) | | **<0.01** | -1.02 | (-1.43 to -0.61) | | **<0.01** | 3.70 | (1.77 to 5.62) | | **<0.01** |
| Rather no | -2.96 | (-3.66 to -2.27) | | **<0.01** | -2.09 | (-2.54 to -1.64) | | **<0.01** | -0.94 | (-1.43 to -0.46) | | **<0.01** | 5.36 | (3.09 to 7.63) | | **<0.01** |
| No | -2.09 | (-2.79 to -1.40) | | **<0.01** | -1.60 | (-2.05 to -1.15) | | **<0.01** | -0.74 | (-1.22 to -0.26) | | **<0.01** | 3.82 | (1.56 to 6.09) | | **<0.01** |
| Sleeping habits |  |  |  |  |  |  |  |  |  |  |  |  |  |  |  |  |
| Less than usual | Ref. | | | | Ref. | | | | Ref. | | | | Ref. | | | |
| Same as usual | -2.26 | (-2.74 to -1.79) | | **<0.01** | -1.71 | (-2.20 to -1.59) | | **<0.01** | -2.60 | (-2.94 to -2.27) | | **<0.01** | 8.56 | (7.00 to 10.11) | | **<0.01** |
| More than usual | -3.74 | -4.23 to -3.26) | | **<0.01** | -2.09 | (-3.34 to -2.72) | | **<0.01** | -4.45 | (-4.79 to -4.12) | | **<0.01** | 20.39 | (18.82 to 21.96) | | **<0.01** |
| Eating habits |  |  |  |  |  |  |  |  |  |  |  |  |  |  |  |  |
| More than usual | 0.26 | (-0.28 to 0.80) | | 0.35 | 0.13 | (-0.22 to 0.47) | | 0.48 | 0.21 | (-0.17 to 0.58) | | 0.28 | 0.45 | (-1.30 to 2.21) | | 0.61 |
| Less than usual | 1.90 | (1.43 to 2.38) | | **<0.01** | 1.21 | (0.90 to 1.52) | | **<0.01** | 2.09 | (1.76 to 2.42) | | **<0.01** | -6.88 | (-8.43 to -5.33) | | **<0.01** |
| Same as usual | Ref. | | | | Ref. | | | | Ref. | | | |  |  |  |  |
| Exercise |  |  |  |  |  |  |  |  |  |  |  |  |  |  |  |  |
| Less than usual | Ref. | | | | Ref. | | | | Ref. | | | | Ref. | | | |
| Same as usual | -0.18 | (-0.61 to 0.25) | | 0.40 | -0.20 | (-0.48 to 0.08) | | 0.16 | -0.41 | (-0.71 to -0.12) | | **0.01** | 2.78 | (1.39 to 4.18) | | **<0.01** |
| More than usual | 0.12 | (-0.42 to 0.65) | | 0.67 | 0.08 | (-0.26 to 0.43) | | 0.65 | -0.36 | (-0.74 to 0.01) | | 0.05 | 3.47 | (1.72 to 5.21) | | **<0.01** |
| Alcohol |  |  |  |  |  |  |  |  |  |  |  |  |  |  |  |  |
| Same as usual | 0.00 | (-0.64 to 0.64) | | 1.00 | -0.06 | (-0.47 to 0.35) | | 0.78 | -0.19 | (-0.64 to 0.25) | | 0.39 | -1.29 | (-3.37 to 0.79) | | 0.22 |
| Less than usual | Ref. | | | | Ref. | | | | Ref. | | | | Ref. | | | |
| More than usual | 1.19 | (0.41 to 1.98) | | **<0.01** | 0.98 | (0.47 to 1.49) | | **<0.01** | 0.89 | (0.35 to 1.44) | | **<0.01** | -4.16 | (-6.73 to -1.60) | | **<0.01** |
| Tobacco |  |  |  |  |  |  |  |  |  |  |  |  | Ref. | | | |
| Same as usual | -0.41 | (-1.39 to 0.57) | | 0.41 | -0.62 | (-1.25 to 0.01) | | 0.05 | -0.39 | (-1.07 to 0.29) | | 0.27 | 0.59 | (-2.60 to 3.79) | | 0.72 |
| Less than usual | Ref. | | | | Ref. | | | | Ref. | | | |  |  |  |  |
| More than usual | 0.44 | (-0.66 to 1.54) | | 0.43 | -0.02 | (-0.73 to 0.69) | | 0.96 | 0.14 | (-0.62 to 0.90) | | 0.71 | -1.78 | (-5.35 to 1.80) | | 0.33 |

**Figure S1.** Flowchart of survey population sampling
